# The influence of oculomotor tasks on postural control in dyslexic children

**DOI:** 10.3389/fnhum.2014.00981

**Published:** 2014-12-09

**Authors:** Maria Pia Bucci, Damien Mélithe, Layla Ajrezo, Emmanuel Bui-Quoc, Christophe-Loic Gérard

**Affiliations:** ^1^UMR 1141, INSERM-Université Paris 7, Hôpital Robert DebréParis, France; ^2^Service d’Ophtalmologie, Hôpital Universitaire Robert DebréParis, France; ^3^Service de Psychopathologie de l’Enfant et de l’Adolescent, Hôpital Universitaire Robert DebréParis, France

**Keywords:** children, dyslexia, eye movements, posture, dual-task

## Abstract

Dual task is known to affect postural stability in children. We explored the effect of visual tasks on postural control in thirty dyslexic children. A selected group of thirty chronological age-matched non-dyslexic children (mean age: 9.92 ± 0.35 years) and a group of thirty reading age-matched non-dyslexic children (mean reading age: 7.90 ± 0.25 years) were chosen for comparison. All children underwent ophthalmologic and optometric evaluation. Eye movements were recorded by a video-oculography system (EyeBrain® T2) and postural sway was recorded simultaneously by a force platform (TechnoConept®). All children performed fixations, pursuits, pro- and anti-saccades tasks. Dyslexic children showed significantly poor near fusional vergence ranges (convergence and divergence) with respect to the non-dyslexic children groups. During the postural task, quality of fixation and anti-saccade performance in dyslexic children were significantly worse compared to the two non-dyslexic children groups. In contrast, the number of catch-up saccades during pursuits and the latency of pro- and anti-saccades were similar in the three groups of children examined. Concerning postural quality, dyslexic children were more unstable than chronological age-matched non-dyslexic children group. For all three groups of children tested we also observed that executing saccades (pro- and anti-saccades) reduced postural values significantly in comparison with fixation and pursuit tasks. The impairment in convergence and divergence fusional capabilities could be due to an immaturity in cortical structures controlling the vergence system. The poor oculomotor performance reported in dyslexic children suggested a deficit in allocating visual attention and their postural instability observed is in line with the cerebellar impairment previously reported in dyslexic children. Finally, pro- or anti-saccades reduce postural values compared to fixation and pursuit tasks in all groups of children tested, suggesting a different influence of visual tasks on postural control according to their attentional demand.

## Introduction

Postural control is a type of motor control that stabilizes the body in space by integrating sensory inputs (visual, vestibular and proprioceptive) about the body’s position with motor outputs to coordinate the action of muscles and keep the body’s center of mass in proper alignment when standing or moving. It grows in parallel with the maturation of the nervous system (Brandt, 2003). In everyday life, attentional resources used to control posture are frequently shared so as to perform other tasks simultaneously; thus postural stability is naturally part of a dual-task (Woollacott and Shumway-Cook, 2002).

Dyslexia is a neurobiological disorder characterized by “a specific and significant impairment in the development of reading skills that is not solely accounted for by mental age, visual acuity problems, or inadequate schooling” (World Health Organization, ICD-10). Different theories have been suggested for explaining the origins of dyslexia: the phonological theory (Ramus, 2003), the auditory (or auditory processing) theory (Tallal et al., 1993; Lehongre et al., 2011), the visual stress theory (Wilkins et al., 2004; Nandakumar and Leat, 2008), the visuo-attentional hypothesis suggested firstly by the Valdois’s group (Bosse et al., 2007) and by our group (Seassau and Bucci, 2013), the superior colliculus theory (Overton, 2008) the cerebellar theory (Nicolson et al., 1999) and finally the magnocellular impairment theory (Galaburda et al., 1985). Recently, several studies were conducted in order to identify the genetic cause of dyslexia (see Carrion-Castillo et al., 2013; Graham and Fisher, 2013; Raskind et al., 2013; Kere, 2014), however, in the current state of research, the etiology of dyslexia remains unsolved, most likely because it has multifactorial origin (see review from Peterson and Pennington, 2012). This lack of certainty about the origin of dyslexia obviously causes difficulties in the care of dyslexic children.

As early as 1973, Frank and Levinson (1973) were the first to make the subjective hypothesis of neurological signs of cerebellar-vestibular deficiency in a dyslexic population thanks to a positive Romberg test. Romberg test is used for testing neurological function: the patient is asked to remove his shoes and stand with his two feet together. The arms are held next to the body; a positive Romberg test is when a swaying and even toppling over occurs (Black et al., 1982). Frank and Levinson (1973) also observed difficulty in tandem walking, articulatory speech disorders, hypotonia, and several dysmetric deficits. Indeed, they reported that 97% of 115 children with dyslexia examined, presented signs in agreement with such hypothesis. The cerebellar deficit hypothesis was confirmed by Nicolson and Fawcett (1999) who noted balance and motor coordination deficits in a population of dyslexic children; as their postural stability was affected by a secondary task, shifting attention away from the primary postural one. These authors suggested that dyslexics needed to invest more attentional resources than non-dyslexics to control their balance when two tasks were performed simultaneously.

Several recent studies explored postural performance in dyslexic subjects while performing a single task as well as a dual-task. They showed different results most likely due to different types of secondary tasks used and/or different postural parameters measured. For instance, Ramus et al. (2003) reported an impaired postural control in dyslexic population, but only in some cases, suggesting that poor postural stability was not strictly correlated with dyslexia but that it could be in relationship with other types of developmental disabilities, such as the visual stress. Poblano et al. (2002) suggested that a dy-synchronization and poor precision of motor coordination could be the cause of poor postural stability in dyslexic population. Stoodley et al. (2005) suggested that several cerebellar impairments and magnocellular immaturity could be at the origin of impaired balance capabilities and Rochelle and Talcott (2006) showed that postural instability is observed more frequently in dyslexic subjects with attention deficit hyperactivity disorder (ADHD) and dyspraxia; such similarity between ADHD and dyslexic children based on cerebellar impairment has been also recently reported by Stoodley (2014). Note, however, that the cerebeller deficit hypothesis is still under debate (see review of Stoodley and Stein, 2013) and that the different results supporting or not a cerebellar deficit are most likely due to different types of cognitive tests used in these studies (Barth et al., 2010; Fernandez et al., 2013).

Pozzo et al. (2006) compared postural stability in 50 dyslexic and 42 non-dyslexic children of about 11 years in four different conditions: bipodal and unipodal; eyes open and closed. They highlighted the role of vision on postural control in dyslexics particularly when the postural task was difficult (unipodal condition). In a dual-task condition, Nicolson and Fawcett (1990) reported that postural stability in dyslexic children was affected during a secondary task which shifts the attention away from posture. They suggested that dyslexics needed to invest more attentional resources than non-dyslexics to control their balance when two tasks were performed simultaneously. On the other hand, Vieira et al. (2009) showed that a cognitive task, such as reading isolated words deteriorated postural stability in dyslexic children. Quercia et al. (2011) showed that dyslexic children were significantly more unstable than normal children when they performed an attentional task such as counting stars projected in front of them on paper. Furthermore, they showed that a vibration at 85 Hz applied to the ankle’ muscles deteriorated stability in dyslexics more than in non-dyslexic children. They concluded that dyslexic children had a deficit of integration of proprioceptive signals. Legrand et al. (2012) compared the postural stability of 18 dyslexic children vs. 18 non-dyslexic children while performing horizontal and vertical saccades and while reading a text silently. They showed an increase in postural instability in both conditions for dyslexic children compared to non-dyslexic children and showed dyslexics were more unstable when reading a text than when performing saccades. Most likely, the attention used in the reading task was probably responsible for the poor postural control in dyslexic children. In two different studies, Bucci et al. (2013a,b) showed in two different studies, the influence of a cognitive task on postural control in dyslexic children. They showed that in the task of naming a simple object postural stability decreased especially in dyslexic children (Bucci et al., 2013b). In the other study they showed that postural stability while performing a modified Stroop test decreased significantly more in dyslexics than in non-dyslexic children (Bucci et al., 2013a). These authors suggested that the postural instability observed in dyslexic children could be due to cerebellar deficits leading to poor automaticity.

Taken together all these findings are in line with the U-shaped non-linear interaction model of Huxhold et al. (2006) suggesting that the type of secondary task can influence postural stability differently; the attention used for the execution of the secondary task may be responsible for shifting attention away from postural control leading to a change in postural sway.

The present study examines the question of whether visual tasks (fixation, pursuits, pro and anti-saccades) can influence postural stability and investigates which types of oculomotor tasks could improve or decrease balance capabilities in dyslexic children vs. non-dyslexic age-matched children.

Our driven hypothesis, based on previously cited works conducted on the dyslexic population, was that, in comparison to control children (non-dyslexic), children with dyslexia would show poor postural control during the dual-task condition when the hard cognitive task was being accomplished in particular in the fixation, pursuits and the anti-saccades tasks; indeed these oculomotor tasks need to focus attention more than the simple pro-saccade task, given the larger cortical and sub cortical circuits that are activated (Leigh and Zee, 2006). Recall also that attentional performances are significantly impaired in dyslexic children (Ruffino et al., 2010) consequently we could expect to find poor postural control during a dual oculomotor task in dyslexic children. On the other hand, based on our previous work (Ajrezo et al., 2013) and according to the model of Huxhold et al. (2006) postural control in children (both dyslexic as well as non dyslexic) could improve while they perform a pro-saccade task as a secondary task.

## Materials and methods

### Subjects

Thirty dyslexic children participated in the study. Dyslexic children were recruited from a pediatric hospital where they had been referred for a complete evaluation of their dyslexia with an extensive examination including neurological/psychological and phonological capabilities. For each child, we assessed the time required to read a passage of text, text comprehension, and the ability to read words and pseudo words were evaluated using the L2MA battery (Chevrie-Muller et al., 1997). This is the standard test developed by the Applied Psychology Center in Paris (*Centre de Psychologie Appliquée de Paris*), and used throughout France. Inclusion criteria were scores on the L2MA which were more than two standard deviations from the mean, and a normal mean intelligence quotient (IQ, evaluated using the WISC-IV), namely between 85 and 115. The mean age of the children with dyslexia was 9.80 ± 0.28 years, the mean IQ was 102.97 ± 1.39, and the mean reading age was 7.80 ± 0.25 years. Children with dyslexia had no sign of hyperactivity or developmental coordination disorder (DCD). A selected chronological age-matched control group (mean age 9.92 ± 0.35 years) of 30 non-dyslexic children and reading age-matched control group of 30 non-dyslexic children (mean reading age 7.90 ± 0.25 years) were chosen for comparison. For reading capabilities the Evaluation de la Lecture en FluencE (E.L.FE) test was used^1^. These children had to satisfy the following criteria: no known neurological or psychiatric abnormalities, no history of reading difficulties or difficulties with near vision. Intelligence quotient was not available for the two groups of non dyslexic children, but their scores for French (reading, comprehension and spelling), mathematics and foreign languages were all above the mean scores for their respective classes. Note that recruitment of controls based on school performance alone has been used by other researchers (Stein et al., 1987; Riddell et al., 1990). Chronological and reading age of the three groups of children tested is shown in Table 1.

**Table 1:** **Chronological and reading age mean values of the three groups of children tested**.

Children group	Chronological age (years)	Reading age (years)
Dyslexic	9.8 [7.4–13.3]	7.8 [6–11]
Non dyslexic reading age-matched	7.5 [6–11.2]	7.9 [6–11]
Non dyslexic chronological age-matched	9.9 [7.1–13.2]	10 [7–13]

*Mean and minimum and maximum values (in square brackets) of chronological and reading age for the three groups of children tested*.

The investigation adhered to the principles of the Declaration of Helsinki and was approved by our institutional Human Experimentation Committee. Informed written consent was obtained for each subject and from the children’s parents after careful review of the experimentation with the participants.

### Visual tasks

Three visual tasks were designed and performed in separate sessions: fixation, pro-saccades and anti-saccades (see Bucci et al., 2014 for details).

*Fixation*: Children had to fixate a white-filled circle subtending a visual angle of 0.5° appearing in the center of the screen and switched on during the postural measurement.

*Pursuits*: The target was moving on the PC’s screen with a linear speed of 15°/s.

*Pro-saccades*: Horizontal, visually-guided saccades were elicited. The stimulus was a red-filled circle subtending a visual angle of 0.5°. The trial consisted of a target positioned at the center of the screen (for a variable delay comprised between 2000 and 3500 ms). After this fixation period, the central target was turned off and a target appeared immediately for 1000 ms to the right or to the left side of the screen. The central fixation target then reappeared, signaling the beginning of the next trial.

*Anti-saccades*: The trial consisted of a target positioned at the center of the screen for a variable delay comprised between 2000 and 3500 ms, followed by its disappearance during a gap interval of 200 ms. Then, a lateral target appeared randomly to the left or to the right of the center, and stayed on for 1000 ms. The central fixation target then reappeared, signaling the beginning of the next trial. Child was instructed to look at the central fixation point, then to trigger a saccade as soon as possible in the opposite direction. When the target returned to the center, the child was instructed to visually follow it back to the center. An initial training block of trials was given to ensure that the instructions were well understood.

The order of visual task presentation was randomly chosen across children to avoid possible fatigue or learning effect.

While performing the visual tasks, child was standing on a platform and both eye movements and posture were recorded simultaneously. The stimuli were presented on a flat PC screen of 22”, its resolution was 1920 × 1080 and the refresh rate was 60 Hz. Each task was performed during 25.6 s.

### Eye movement recording

Eye movements were recorded by Mobile EyeBrain Tracker (Mobile T2®, e(ye)BRAIN).^2^ A calibration was done before starting the experiment (see Lions et al., 2013 for details).

### Postural recording

To measure postural stability, we used a platform (principle of strain gauge) consisting of two dynamometric clogs (Standards by *Association Française de Posturologie*, produced by TechnoConcept, Céreste, France). The excursions of the center of pressure (COP) were measured during 25.6 s; the equipment contained a 16-bit analog-digital. The sampling frequency of the COP was 40 Hz.

Postural measurements were performed in Standard Romberg condition: heels were placed 4 cm apart and feet positioned symmetrically with respect to the child’s sagittal axis at a 30° angle. Before running postural measure for each child, the program asked to add weight, height and shoe size. Postural analysis takes in account these individual data.

For each visual task two postural recordings were done successively. The experimental sessions took place in a dark room to avoid children fixating other stimuli. Children were placed 60 cm away from the screen, where visual tasks were presented at eye level. Children were asked to stand without moving their body and with their arms along their body. Children were asked not to move their head during the visual tasks.

### Data processing

Eye movements were analyzed using the better signal of both eyes, that was the right eye for the majority of children tested (90%, 88% and 92% for dyslexic, reading age-matched and chronological age-matched children group, respectively). During the fixation task, the number of intrusive saccades with amplitude ≥2° was counted. It is well known that microsaccades are normally smaller than such amplitudes (Krekelberg, 2011). For pursuit movements, the number of catch-up saccades was measured (saccades made in the pursuit direction, with amplitude ≥ ± 2°). For each saccade recorded during the pro- and anti-saccades tasks, we examined the latency value in milliseconds (i.e., time needed to prepare and trigger the saccades). Furthermore, in the anti-saccade task, the mean error rate was also examined (i.e., the ratio between the number of saccades made in the wrong direction in relation to the lateral target and the total number of saccades made in the target direction).

The MeyeAnalysis© software (provided with the eye tracker^3^) was used to determine automatically the onset and the end of each saccade by using a “built-in saccade detection algorithm”. All detected saccades are verified by the investigator and corrected or discarded as necessary (see Bucci and Seassau, 2012).

To quantify the effect of visual tasks on the postural performance, three parameters of the platform recording were analyzed: surface area, length and mean speed of the CoP.

### Statistical analysis

For clinical data on fusional vergence values analysis of variance (ANOVA) was performed with groups of children as inter-subject factor and divergence and convergence values as within subject factor. Mixed-design multivariate analysis of variance (MANOVA) tests were conducted to analyze differences in dual task between the three groups of children. Furthermore, in order to explore the different effects of the different types of eye movements on postural parameters analysis of variance ANOVAs were performed with repeated measures. *Post hoc* comparisons were made with the Fischer’s least significant differences (LSD). The effect of a factor was considered as significant when the *p*-value was below 0.05.

## Results

### Visual evaluation

**Figure 1:**
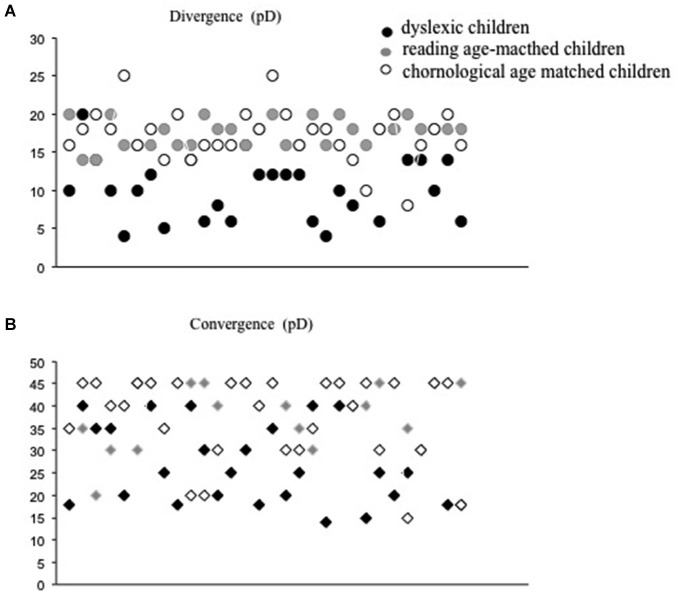
**Divergence (A) and convergence values in prism dioptres (B) for each child tested (dyslexic, chronological and reading age-matched)**.

All children tested underwent ophthalmological examination accompanied by optometric evaluation of their visual functions (mean values shown in Table 2). After subjective refraction, the monocular visual acuity was normal (≥20/20) for all children tested. All children had normal binocular vision, as evaluated with the Netherlands Organization of Applied Scientific Research Test of stereoacuity (TNO). Near point of convergence was normal for the three groups of children tested (≤5 cm). In addition, an evaluation of fusional vergence capability using prisms bar at near distance was performed. Phoria, which is defined as deviation kept latent by the fusion mechanism (Von Noorden and Campos, 2002) was measured by the cover-uncover test for the three groups and it was similar in three groups of children tested. The fusional divergence and convergence ranges were significantly smaller in the dyslexic group with respect to the other two groups of non dyslexic children (see Figure 1). The Analysis of variance showed a significant main effect of group (*F*_(2,87)_ = 26.48, *p* < 0.0001, eta squared = 0.32 and *F*_(2,87)_ = 10.43, *p* < 0.0001, eta squared = 0.35, for divergence and convergence amplitude, respectively).

**Table 2:** **Clinical characteristics of the three groups of children examined**.

Children group	TNO (sec of arc)	NPC (cm)	Heterophoria (pD)	Fusional convergence ranges (pD)	Fusional divergence ranges (pD)
Dyslexic	69 ± 7.8	3.2 ± 0.5	−2.2 ± 0.7	29.1 ± 1.8	10.46 ± 0.8
Non-dyslexic reading age-matched	65 ± 6	3 ± 0.3	−3 ± 0.6	39 ± 1*	17.5 ± 0.3*
Non-dyslexic chronological age-matched	68 ± 6.7	3.0 ± 0.6	−3.3 ± 0.7	36.9 ± 1.8*	17.93 ± 1.1*

Clinical characteristics of all children tested. Mean and standard error values for binocular vision (stereoacuity test, TNO measured in seconds of arc); near point of convergence (NPC measured in cm); heterophoria at near distance, measured in prism diopters (pD); fusional vergence ranges (divergence and convergence) at near distance, measured in prism diopters (pD). * Value is significantly different from the dyslexic group of children.

### Dual task

Postural stability for the three different groups of children showed a significant group effect while children were performing fixation task (Pillai-Bartlett trace 0.59, *F*_(26,130)_ = 2.30, *p* < 0.01) and pro-saccades task (Pillai-Bartlett trace 0.52, *F*_(26,130)_ = 1.73, *p* < 0.02). The group of chronological age-matched children showed a better stability with respect to the other two groups of children (dyslexic and non dyslexic age-matched children). In order to point out the different effect of the visual tasks on each postural parameter ANOVA was run on each measure.

### Eye movements

**Figure 2:**
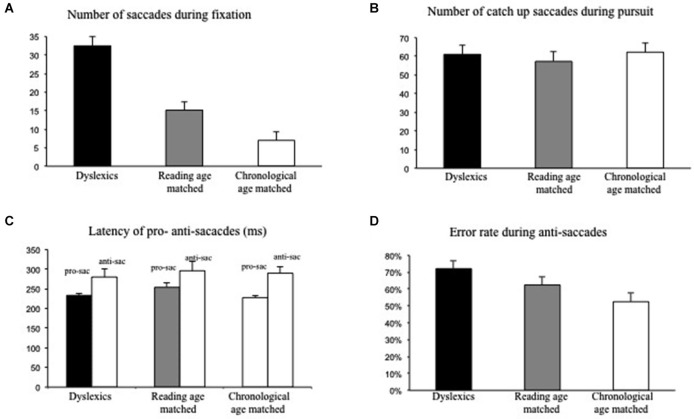
**Eye movements recorded during postural task for both groups of children tested (dyslexic and non-dyslexic children). (A)** Mean values of number of intrusive saccades during fixation. **(B)** Mean values of number of catch-up saccades during pursuits. **(C)** Mean values of latency (in ms) of pro- and anti-saccades. **(D)** Mean error rate in anti-saccades (in percentage). Vertical bars indicate the standard error.

Figure 2A shows the mean number of intrusive saccades during fixation for each group of children. The number of intrusive saccades during fixation was significantly larger for dyslexic children with respect to the other two groups of non dyslexic children. Analysis of variance showed a significant group effect (*F*_(2,87)_ = 16.16; *p* < 0.01, eta squared = 0.2); *post hoc* comparisons showed that the number of saccades during fixation task was significantly higher in dyslexic children with respect to reading age-matched children (*p* < 0.01) and to chronological age-matched children (*p* < 0.001).

Figure 2B shows the mean number of catch-up saccades recorded during pursuits. The number of catch-up saccades was similar in the three groups of children tested. Analysis of variance did not show a significant group effect (*F*_(2,87)_ = 0.15; *p* = 0.8).

Figure 2C shows the mean latency of pro- and anti-saccades. The three groups of children did not show any difference in the latency of pro- and anti-saccades; ANOVA failed to show a significant group effect (*F*_(1,87)_ = 0.19; *p* = 0.8), but only a significant effect of the task: latency values of anti-saccades were significantly longer than those of pro-saccades (*F*_(1,87)_ = 17.92; *p* < 0.01, eta squared = 0.2).

Finally, Figure 2D shows the error rate observed during the anti-saccade task. The error rate was significantly higher in dysleixc children; ANOVA showed a significant effect of group (*F*_(2,87)_ = 4.25; *p* < 0.01 eta squared = 0.2); *post hoc* comparisons showed that the error rate in dyslexic children was significantly higher with respect to chronological age-matched children group only (*p* < 0.01).

### Postural control

**Figure 3:**
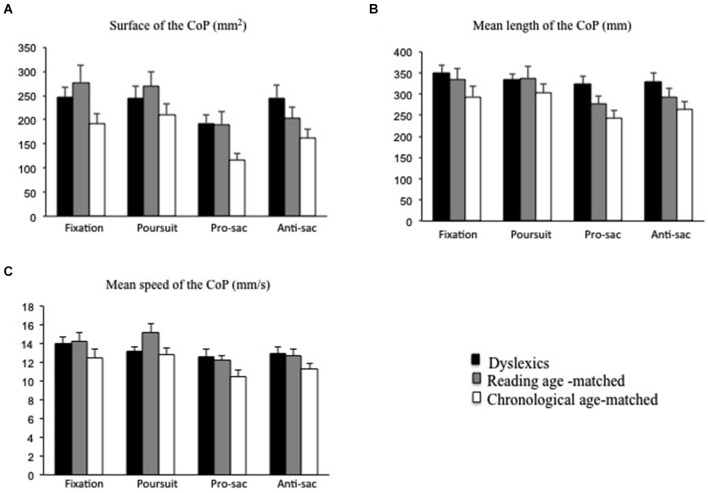
**Postural parameters recorded during fixation, pursuits, pro- and anti-saccades for both groups of children tested (dyslexic and non-dyslexic children). (A)** Mean values of the surface of the CoP (in mm^2^) during fixation, pursuits, pro- and anti-saccades. **(B)** Mean values of the length of the CoP (in mm) during fixation, pursuits, pro- and anti-saccades. **(C)** Mean values of the mean speed of the CoP (in mm/s) during fixation, pursuits, pro- and anti-saccades. Vertical bars indicate the standard error.

Figure 3A shows the mean surface of the CoP for the three groups of children tested during fixation, pursuits, pro- and anti-saccades tasks. The mean value of the surface of the CoP for dyslexic children was larger with respect to those reported in chronological age-matched children. The ANOVA showed a significant group effect (*F*_(2,87)_ = 3.26; *p* < 0.01, eta squared = 0.07). *Post hoc* comparisons showed that the surface of the CoP in dyslexic children was significantly higher with respect to chronological age-matched children group only (*p* < 0.01).

Moreover, ANOVA showed a significant effect of the visual task (*F*_(3,261)_ = 12;36 ; *p* < 0.01, eta squared = 0.13). *Post hoc* comparisons showed that the mean value of the surface of the CoP was significantly smaller during pro-saccades with respect to fixation (*p* < 0.01), pursuits (*p* < 0.01) and anti-saccades (*p* < 0.01).

Figure 3B shows the mean values of the length of the CoP for each group of children during fixation, pursuits, pro- and anti-saccades tasks. Analysis of variance showed a significant group effect (*F*_(2,87)_ = 4.19; *p* < 0.04, eta squared = 0.07). *Post hoc* comparisons showed that the length of the CoP was significantly larger in dyslexic children with respect to chronological age-matched children group only (*p* < 0.01).

Furthermore, ANOVA showed a significant effect of the visual task (*F*_(3,261)_ = 8.85, *p* < 0.01, eta squared = 0.10). *Post hoc* comparisons showed that the mean value of the length of the CoP was significantly lower during pro-saccades in comparison to fixation (*p* < 0.01) and pursuits (*p* < 0.01); similarly, the mean value of the length of the CoP was significantly lower in the anti-saccades that in fixation (*p* < 0.02) and pursuits (*p* < 0.05).

Figure 3C shows the mean value of the mean speed of the CoP for the three groups during fixation, pursuits, pro- and anti-saccades tasks. The mean speed of the CoP was similar in the three groups of children tested. Analysis of variance failed to show a significant group effect (*F*_(2,87)_ = 1.71; *p* = 1.18). However, ANOVA showed a significant effect of the visual task (*F*_(3,261)_ = 10.74; *p* < 0.01, eta squared = 0.11). *Post hoc* comparisons showed that the mean value of the mean speed of the CoP during the pro-saccades task was significantly smaller to that of fixation and pursuits (*p* < 0.01 and *p* < 0.01, respectively for fixation and pursuits); similarly, the mean value of the mean speed of the CoP was significantly smaller in the anti-saccades that in fixation (*p* < 0.01) and pursuits (*p* < 0.05).

## Discussion

The main findings of this study are as follows: (i) dyslexic children showed significantly poor near fusional vergence ranges (convergence and divergence) with respect to non-dyslexic children groups; (ii) during the postural task: the quality of fixation and the anti-saccades performance in dyslexic children were significantly worse than in non-dyslexic children. In contrast, the number of catch-up saccades during pursuits and the latency of pro- and anti-saccades were similar in the three groups of children examined; (iii) dyslexic children were more unstable than chronological age-matched non-dyslexic children; (iv) in all three groups of children tested executing saccades (pro- and anti-saccades) improved postural control significantly in comparison with fixation and pursuit tasks. These findings will be discussed individually below.

### Poor vergence fusional capabilities in dyslexic children

According to previous studies vergence fusional capabilities are poor in dyslexic children (Hung, 1989; Buzzelli, 1991; Bucci et al., 2013a,b). These results suggest a general immaturity of the cortical structures controlling the vergence system in dyslexic children. Indeed, fusional vergence capabilities are age dependent (Scheiman et al., 1989; Palomo Alvarez et al., 2006) and recent studies showed evidence of vergence control at the cortical level. For instance, Quinlan and Culham (2007) and also Alkan et al. (2011) in a fMRI study showed an activation of parietal, occipital cortex and also of the frontal eye fields and midbrain while humans performed convergence.

We suggest that orthoptic vergence training could be applied for dyslexic children in order to improve their vergence capabilities given that previous studies showed an improvement of vergence fusional amplitude in children with vergence insufficiency (Bucci et al., 2004; Scheiman et al., 2005).

### Oculomotor performance during dual-task in dyslexic children

We found that the quality of fixation during dual-task in dyslexic children was significantly worse in comparison with the two groups of non-dyslexic children. This finding could be related to visual attention deficits reported in dyslexic children and their difficulty to inhibit unwanted saccades during a fixation task.

On the other hand, the error rate when performing anti-saccades tasks was significantly higher in dyslexic children than in chorological age-matched non-dyslexic children. This finding was also reported in a previous study from Bucci et al. (2012) where dyslexics executed a simple visual anti-saccades task, i.e., while they were seated comfortably on a chair and postural control was not measured.

Nonetheless, we observed during the dual-task that the performance of pursuits and the latency of saccades (pro- and anti-saccades) were similar in the dyslexic and non-dyslexic children groups. This result is in agreement with a previous study of Bucci et al. (2014) examining saccade performance in children with ADHD (treated or not with methylphenidate) during the dual-task condition. These authors also showed that during the dual-task, the performance of pursuits and saccades (pro- as well as anti-saccades) were similar in children with ADHD (off and on methylphenidate) and healthy children.

These results suggest that while dyslexic children are in the dual-task condition, they focus more on the visual task than on the postural task. Indeed, Bucci et al. (2012) reported that in simple oculomotor task both the latency of pro- and anti-saccades as well as the error rate during anti-saccades is significantly different in dyslexic children with respect to non dyslexic children, that is dyslexics showed longer latency and a high error rate of anti-saccades.

### Postural stability is poor in dyslexic children

Our study showed that dyslexic children were more unstable than chronological age-matched non-dyslexic children. This result is line with several works on dyslexic children during a dual-task by our group (e.g., Legrand et al., 2012; Bucci et al., 2013a,b) and also other authors (Pozzo et al., 2006; Vieira et al., 2009; Quercia et al., 2011); together all these studies supported the hypothesis of a cerebellar deficit in dyslexic children suggested by Frank and Levinson (1973) first, and subsequently by Nicolson and Fawcett (1999) even if such hypothesis is not shared by all researchers (as said in Section Introduction). Recall that neurophysiological studies also confirmed such thinking; for instance Rae et al. (1998) have found in dyslexic adults biomechanical lateral differences in the temporo-parietal lobes of the cerebellum that were not present in non-dyslexic adult subjects. An MRI study by Eckert et al. (2003) also found smaller right anterior lobes of the cerebellum in dyslexic children with respect to non-dyslexic children. Pernet et al. (2009) reported also that the right cerebellar and the right lentiform nucleus were two areas that maximally differ between control and dyslexic adults.

Thus, dyslexic children are not able to use all sensory input correctly in order to ensure good postural control, such difficulty is particularly observed while these children are asked to perform a dual-task. This hypothesis is in relation to the study of Barela et al. (2011) which suggest that the different performance observed in dyslexic children during motor tasks could be due to difficulties in coupling sensory information (e.g., visual information) and motor activity (e.g., maintaining postural stability). These authors showed that the coupling between visual and motor information is lower and more variable in dyslexic children compared to non-dyslexic children. Finally we have to point out that postural instability reported in dyslexic children was similar to those observed in the group of reading age-matched children (younger children). This finding supports the hypothesis of an general immaturity of motor control in dyslexic children.

### Oculomotor tasks affect postural sway

The effect of oculomotor tasks on postural control is still controversial and few studies have recorded eye movements and postural in children simultaneously. Our results show that performing pro-saccades improves postural stability with respect to a simple fixation task and pursuit tasks in dyslexic as well as non-dyslexic children. This finding is in line with the report of Ajrezo et al. (2013) showing in a large sample of 95 healthy children a decrease in postural sway as children performed pro-saccades with respect to the fixation task.

The novelty here is that that performing pro-saccades improves postural stability compared not only to fixation but also to pursuits, and this occurs in all groups of children tested. This result is in line with the recent study of Bucci et al. (2014) showing also that in healthy children and children with ADHD (off and on methylphenidate), postural stability increased while children executed pro-saccades compared to a pursuit task.

Interestingly, we found that only the value of the length and the mean speed of the CoP is significantly smaller while children perform anti-saccades task compared with fixation and pursuit tasks. This finding is only apparently in contrast with the previous study by Legrand et al. (2013) conducted in young healthy adults. These authors showed that anti-saccades increased the value of the length and the mean speed of the CoP with respect to pro-saccades tasks. This difference is probably due to the fact that Legrand et al. (2013) did not record eye movements at the same time as posture; therefore we cannot be sure that subjects really executed pro- or anti-saccades. This suggests the importance of recording both eye movements and postural sway simultaneously.

The difference effect of pro and anti-saccades on the length and the mean speed of the CoP could be due to the fact that the cortical circuits implied in the programming and executing anti-saccades are broader than those of pro-saccades (Leigh and Zee, 2006). Indeed, more cognitive processes are necessary to realize anti-saccade task because in order to correctly perform an anti-saccade, the reactive saccade toward the peripheral stimulus must be inhibited and a voluntary saccade in the opposite direction (that is to say an anti-saccade) must be programmed.

Finally, our results showed that oculomotor tasks could either increase or decrease postural stability, depending on the type of task and its attentional demand according to the U-shaped non-linear interaction model, described by Huxhold et al. (2006). Indeed, in the case of our study, fixation and pursuit movements are quite difficult attention-demanding tasks leading to degradation of the postural sway. In contrast, an easy task, such as pro-saccades, shifts the attentional focus away from postural control, leading to a better automatic postural performance. The different effect of anti-saccades with respect to pro-saccades on postural control is not observed in all postural parameters measured. This suggests the importance of measuring several postural parameters by using both temporal and spatial analysis of the CoP in order to improve our understanding of the effect of oculomotor tasks on postural control.

## Conclusion

The poor oculomotor performance reported in dyslexic children during a dual-task suggests a deficit in allocating visual attention and their postural instability observed is in line with the cerebellar impairment previously suggested in dyslexic children. Finally, executing saccades increases postural stability in both groups of children tested, suggesting a different influence of visual tasks on postural control according to its attentional demand.

## Conflict of interest statement

The authors declare that the research was conducted in the absence of any commercial or financial relationships that could be construed as a potential conflict of interest.

## Footnotes

^1^http://www.cognisciences.com

^2^www.eye-brain.com

^3^See www.eye-brain.com
